# Heterogeneity in the links between sleep arousals, amyloid-**β**, and cognition

**DOI:** 10.1172/jci.insight.152858

**Published:** 2021-12-22

**Authors:** Daphne O. Chylinski, Maxime Van Egroo, Justinas Narbutas, Martin Grignard, Ekaterina Koshmanova, Christian Berthomier, Pierre Berthomier, Marie Brandewinder, Eric Salmon, Mohamed Ali Bahri, Christine Bastin, Fabienne Collette, Christophe Phillips, Pierre Maquet, Vincenzo Muto, Gilles Vandewalle

**Affiliations:** 1 GIGA-Cyclotron Research Centre-In Vivo Imaging and; 2 Psychology and Cognitive Neuroscience Research Unit, University of Liege, Liege, Belgium.; 3 Physip SA, Paris, France.; 4 Department of Neurology, University Hospital of Liege, Liege, Belgium.; 5 GIGA-In Silico Medicine, University of Liege, Liege, Belgium.

**Keywords:** Aging, Neuroscience, Alzheimer disease

## Abstract

**BACKGROUND:**

Tight relationships between sleep quality, cognition, and amyloid-β (Aβ) accumulation, a hallmark of Alzheimer’s disease (AD) neuropathology, have been shown. Sleep arousals become more prevalent with aging and are considered to reflect poorer sleep quality. However, heterogeneity in arousals has been suggested while their associations with Aβ and cognition are not established.

**METHODS:**

We recorded undisturbed night-time sleep with EEG in 101 healthy individuals aged 50–70 years, devoid of cognitive and sleep disorders. We classified spontaneous arousals according to their association with muscular tone increase (M+/M–) and sleep stage transition (T+/T–). We assessed cortical Aβ burden over earliest affected regions via PET imaging and assessed cognition via neuropsychological testing.

**RESULTS:**

Arousal types differed in their oscillatory composition in θ (4–8 Hz) and β (16–30 Hz) EEG bands. Furthermore, T+M– arousals, interrupting sleep continuity, were positively linked to Aβ burden (*P* = 0.0053, *R²*_β_* = 0.08). By contrast, more prevalent T–M+ arousals, upholding sleep continuity, were associated with lower Aβ burden (*P* = 0.0003, *R²*_β_* = 0.13), and better cognition, particularly over the attentional domain (*P* < 0.05, *R²*_β_* ≥ 0.04).

**CONCLUSION:**

Contrasting with what is commonly accepted, we provide empirical evidence that arousals are diverse and differently associated with early AD-related neuropathology and cognition. This suggests that sleep arousals, and their coalescence with other brain oscillations during sleep, may actively contribute to the beneficial functions of sleep and constitute markers of favorable brain and cognitive health trajectories.

**TRIAL REGISTRATION:**

EudraCT 2016-001436-35.

**FUNDING:**

FRS-FNRS Belgium (FRSM 3.4516.11), Actions de Recherche Concertées Fédération Wallonie-Bruxelles (SLEEPDEM 17/27-09), ULiège, and European Regional Development Fund (Radiomed Project).

## Introduction

Sleep is central to health and cognition, and it deteriorates with aging (1). In addition, sleep disruption is associated with Alzheimer’s disease (AD), as assessed by hyperphosphorylated tau and amyloid-β (Aβ) brain accumulation, most likely in a bidirectional manner (2, 3). Poorer sleep quality (4), daytime sleepiness (5), reduced slow-wave sleep (6, 7), and sleep deprivation (8, 9) have been linked to higher Aβ levels, but they have also been linked to poorer cognitive performance (7, 10).

Sleep arousals, defined as transient accelerations in sleep electroencephalogram (EEG) rhythms, are usually considered as brain reactions to internal (e.g., apnea) or external (e.g., auditory stimulus) perturbations (11). Although they are key elements of sleep microstructure, they can also shape its macrostructure and lead to a shallower sleep stage (12). Arousals are most often considered as markers of sleep disruption, thereby a detrimental and harmful sleep feature. Several conceptual definitions classified them almost exclusively in the context of sleep disorders (e.g., sleep disordered breathing [SDB] and, to a smaller extent, periodic limb movements syndrome [PLMS]) or, in experimental protocols, inducing arousals through external — mainly auditory — stimulation (13, 14). These types of studies yielded mixed results. A negative link between arousal prevalence during sleep and cognitive performance was revealed in SDB, particularly in attention, and sometimes in the executive and memory domains (15). By contrast, other investigations did not find such a relationship and imputed alterations in cognition in SDB to brain hypoxia (see ref. 15 for review). In individuals devoid of sleep pathologies, arousals evoked by auditory stimuli were reported to impact subsequent daytime alertness (16). In addition, sleep fragmentation induced by auditory stimulation is associated with higher Aβ cerebrospinal fluid (CSF) content the following day (6).

Importantly, spontaneous arousals — i.e., not elicited by any identifiable internal or external stimuli — also constitute an authentic element of undisturbed sleep in healthy individuals. Their mechanisms, cerebral correlates, and functional consequences remain largely unknown (11), with some authors suggesting that there may be physiologic and pathologic arousals. Understanding their respective roles might shed light on the adaptive properties of the sleeping brain and provide insight into pathological mechanisms associated with sleep disturbances (11).

Here, we assessed whether different types of spontaneous arousals during sleep were differentially associated with Aβ cortical deposition and cognitive performance in a cohort of healthy individuals in late midlife. We were able to tease apart different types of arousals, based on their temporal relationships with increased muscular tone and sleep stage transitions. In line with the hypothesis that arousals perturb sleep, we anticipated that arousals fragmenting sleep structure would be associated with both worse cognitive performance and Aβ deposition in brain areas that are first affected by this AD-related neuropathological process.

## Results

### EEG oscillations differ across arousal types.

**Figure 1:**
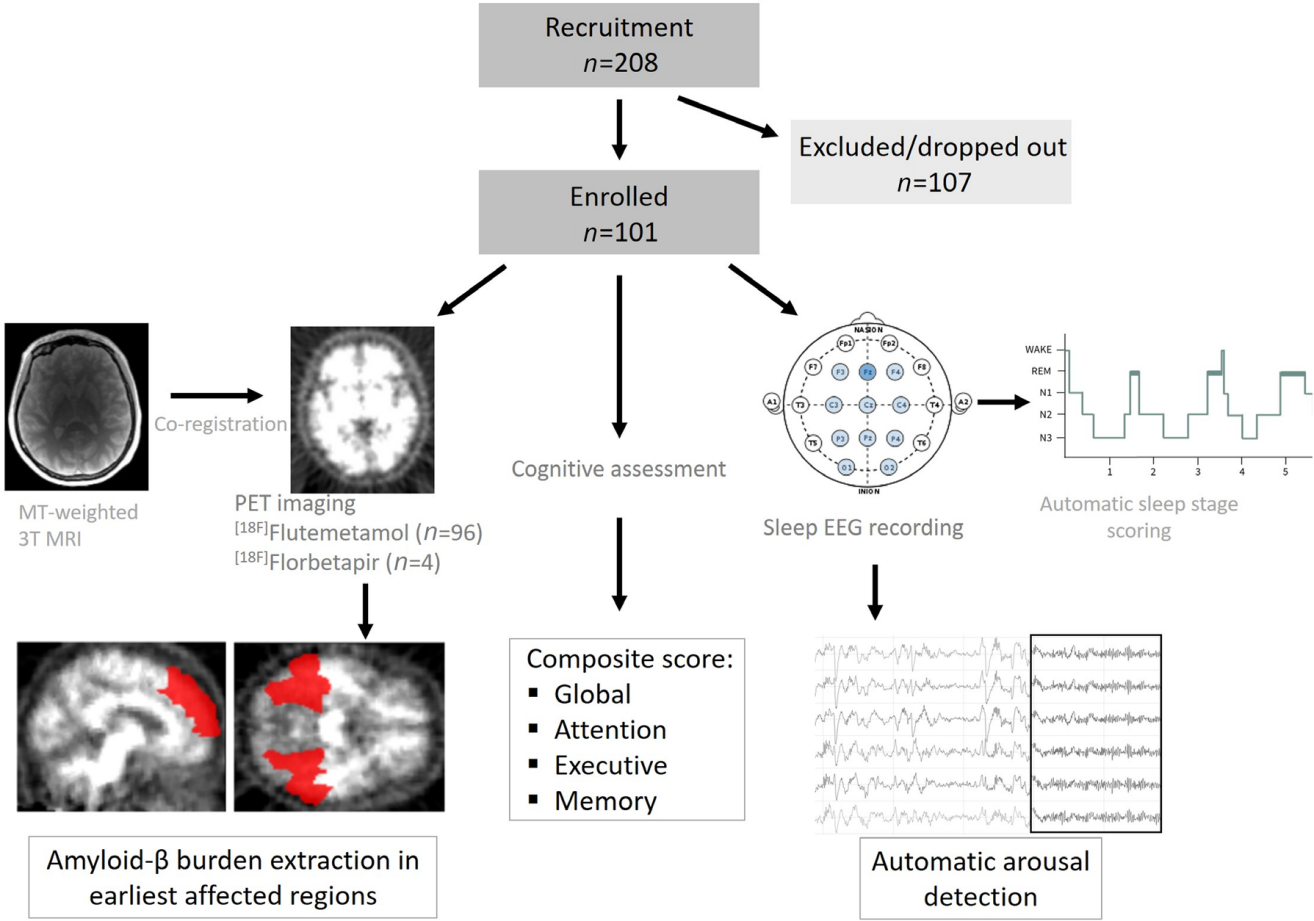
Overview of the study. In total, 208 participants were recruited, of which 107 did not participate in the study, as they were excluded based on inclusion criteria (see Methods), if sleep apnea were detected (>15/hour) or decided to withdraw. Participants underwent [^18^F]Flutemetamol (*n* = 96)/[^18^F]Florbetapir (*n* = 4) PET scan to assess Aβ burden, which we extracted over the earliest affected regions; they were also tested via an extensive battery of neuropsychological tasks from which we extracted global score, as well as performance over 3 main cognitive domains (attention, executive, and memory); and habitual sleep was recorded via EEG from which arousals were automatically detected.

We recorded undisturbed sleep at habitual sleep times under EEG in 101 healthy individuals aged 50–70 years (59 ± 5 years; 68 women), following 1 week of regular sleep-wake schedule (Figure 1). In order to evaluate the potential heterogeneity of arousals, we split them according to 2 criteria, which we considered as relevant in research settings, as well as in clinical practice. We first chose to focus on whether arousals did trigger a sleep stage transition (T+) (when they occurred within 15 seconds [15s] of a stage change) or not (T–), as arousals may or may not lead to a lighter sleep stage (12) and they have already been investigated in that regard (17). Secondly, we considered their salience, reflected by the concomitant increase in electromyogram (EMG) tone (M+) or its absence (M–), as it is among the arousal scoring criteria (needed to score an arousal in rapid eye movement [REM] sleep).

**Figure 2:**
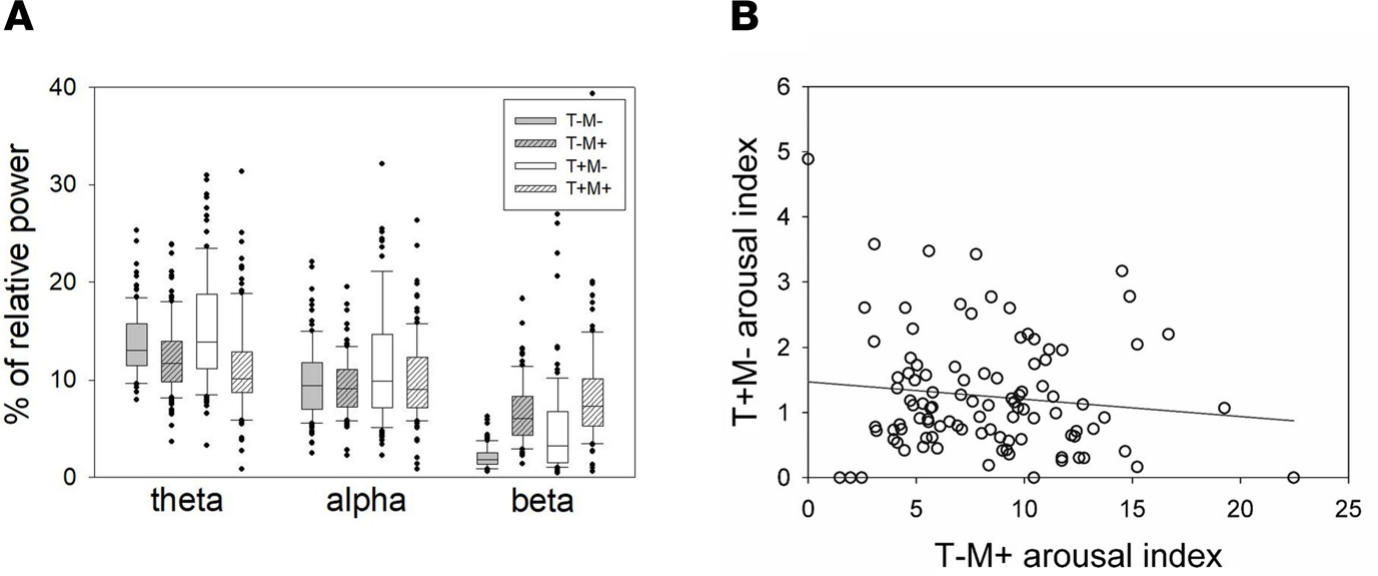
Spectral composition of arousal types. (**A**) Box plot of relative power in the θ (4.5–7.5 Hz), α (8.5–11.5 Hz), and β (16.5–29.5 Hz) band for T–M–, T–M+, T+M–, and T+M+ arousals with error bars. The boxes’ central lines indicate the median of power values, with the bottom and upper edges showing the 25th and 75th percentiles, respectively. T, arousal associated (T+) or not (T–) with sleep stage transition; M, arousal associated (M+) or not (M–) with an increase in EMG tone. Indexes correspond to hourly prevalence. (**B**) Absence of significant correlation between T–M+ arousals and T+M– arousals (Spearman’s *r* = –0.05; *P* = 0.60).

In a first step, we assessed whether characterizing sleep arousals by their association with sleep stage transition (T+ or T–), and the cooccurrence of an EMG tone increase (M+) or not (M–) resulted in differences in their oscillatory properties. We computed individual relative power in the different EEG frequency bands defining an arousal (θ, 4.5–7.5 Hz; α, 8.5–11.5 Hz; and β, 16.5–29.5 Hz). A generalized linear mixed model (GLMM) with relative power as a dependent variable (and adjusted for data distribution) first indicated that relative power changed across frequency bands (F_2,294.1_ = 403.84, *P* < 0.0001, semipartial *R*² [*R*²β*] = 0.73). More importantly, it yielded a triple interaction between transition, EMG status, and frequency band (F_2,880_ = 3.39, *P* = 0.034, *R*²β* = 0.008), implying that arousals differ in their spectral composition based on the presence or absence of EMG changes and sleep stage transition (Figure 2A). This was further reflected in main effects of EMG status (F_1,989.9_ = 75.97, *P* = 0.0001, *R*²β* = 0.07) and transition (F_1,708.6_ = 39.17, *P* < 0.0001, *R*²β* = 0.05), as well as in interactions between transition and frequency band (F_2,709.5_ = 34.62, *P* < 0.0001, *R*²β* = 0.09), between EMG status and frequency band (F_2,979,2_ = 187.39, *P* < 0.0001, *R*²β* = 0.28), and between EMG status and transition (F_1,879.3_ = 22.55, *P* < 0.0001, *R*²β* = 0.025). Based on this first analysis, we therefore concluded that the factors of arousal heterogeneity eloquently define 4 types of arousals (T+M+, T+M–, T–M+, and T–M–). We finally note that multiple post hoc comparisons within each band yielded significant differences across arousal types over the θ and β bands (see Table 1).

### Arousal heterogeneity reflects different associations with Aβ burden.

**Figure 3:**
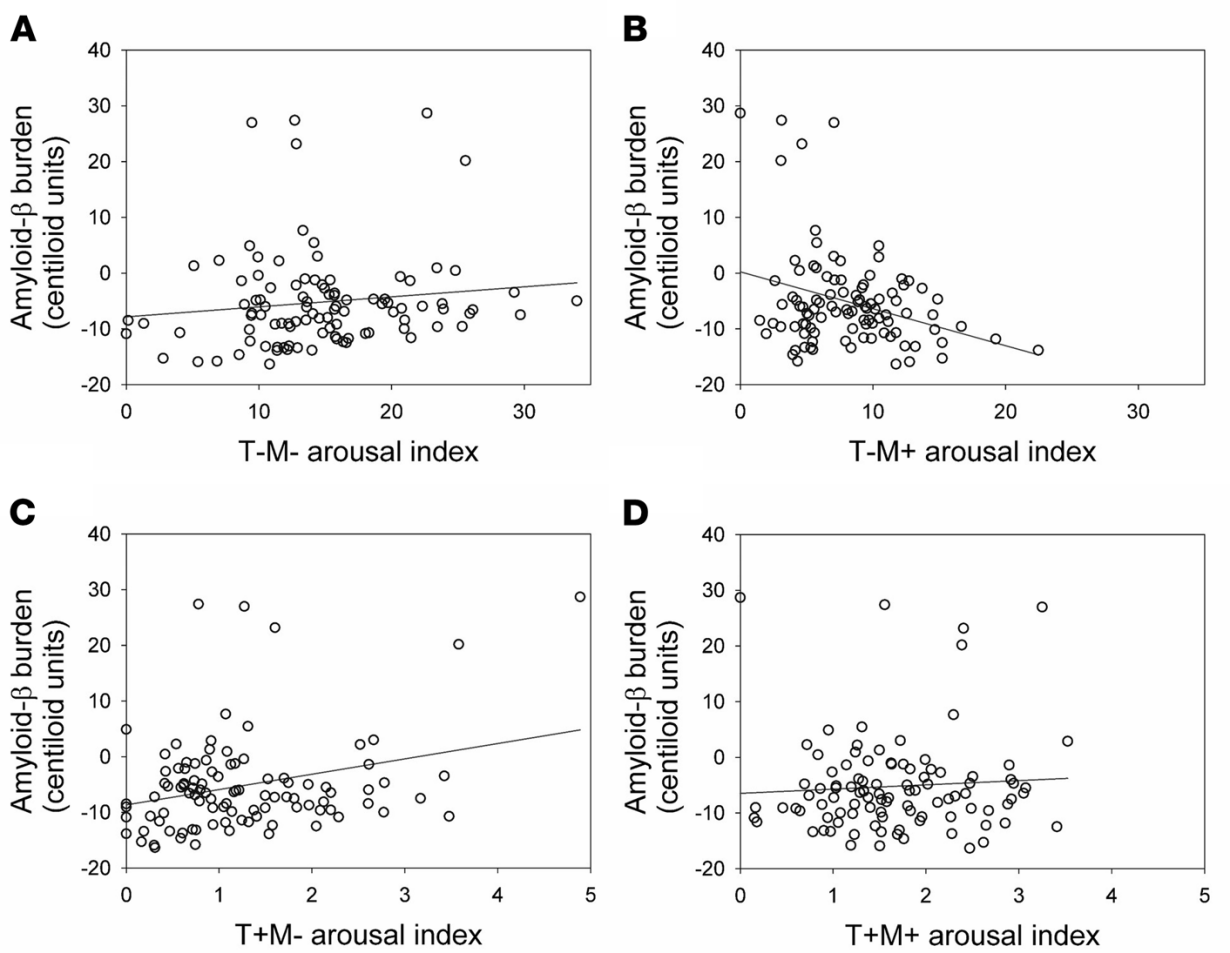
Associations between prevalence of different types of arousals and early cortical Aβ burden. (**A**) No correlation between T–M– arousals and Aβ burden (Spearman’s *r* = 0.16; *P* = 0.11); (**B**) significant negative correlation between T–M+ arousals and Aβ burden (Spearman’s *r* = –0.16, *P* = 0.11); (**C**) significant positive correlation between T+M– arousals and Aβ burden (Spearman’s *r* = 0.17, *P* = 0.08); and (**D**) no correlation between T+M+ arousals (Spearman’s *r* = 0.07, *P* = 0.46). See main text for full GLMM output controlling for several covariates. T, arousal associated (T+) or not (T–) with sleep stage transition; M, arousal associated (M+) or not (M–) with an increase in EMG tone. Indexes correspond to hourly prevalence.

In line with our goal to consider very early AD-related neuropathological process, Aβ burden was quantified over the regions previously reported as the earliest cortical aggregation sites (18) — the frontal medial cortex and basal part of temporal lobe (fusiform and inferior temporal gyri) in all but 1 participant. We tested in a GLMM whether associations between arousal density varied with transition (T+, T–) and EMG (M+, M–) statuses and were associated with Aβ burden, while regressing out age and sex effects. We first observed a main effect of transition (F_1,196_ = 607.52, *P* < 0.0001, *R*²β* = 0.76) and EMG status (F_1,98_ = 70.63, *P* < 0.0001, *R*²β* = 0.42), as well as an interaction between EMG status and transition (F_1,196_ = 102.32, *P* < 0.0001, *R*²β* = 0.34), indicating that density of arousal types significantly varied. Interestingly, we did not find any significant main effect of early cortical Aβ burden (F_1,96_ = 0.26, *P* = 0.61), age (F_1,96_ = 2.44, *P* = 0.12), or sex (F_1,96_ = 0.12, *P* = 0.73). Critically, the GLMM yielded a significant triple interaction between early cortical Aβ burden, EMG status, and transition (F_1,196_ = 7.16, *P* = 0.008, *R*²β* = 0.035), implying that the association between arousals and early cortical Aβ burden depends on the concomitant change in muscular tone and sleep stage transition. The heterogeneity in spontaneous arousals was further reflected by the significant interactions between early cortical Aβ burden and EMG status (F_1,98_ = 8.64, *P* = 0.004, *R*²β* = 0.08). Figure 3 decomposes the associations between each of the 4 types of arousals and early cortical Aβ burden.

We further computed a GLMM with early cortical Aβ burden as a dependent variable to explore whether its association with T–M+ and T+M– arousal truly differed in the part of Aβ burden variance T–M+ and T+M– arousals explained in a more complex model, regressing out age and sex. Both associations were significant with a negative link between Aβ and T–M+ arousals (F_1,95_ = 14.15, *P* = 0.0003, *R*²β* = 0.13) and with a positive association between Aβ and T+M– arousals (F_1,95_ = 8.16, *P* = 0.0053, *R*²β* = 0.08) — together with an expected main effect of age (F_1,95_ = 13.02, *P* = 0.0005, *R*²β* = 0.12) (19) and no main effect of sex (F_1_,_95_ = 2.54, *P* = 0.11). Critically, a post hoc contrast showed that the links between the 2 types of arousals and early cortical Aβ burden were significantly different (t_93_ = 3.73, *P* = 0.0003). In addition, T–M+ and T+M– arousals were not correlated (Figure 2B). Supplementary analysis showed the same statistical picture in a GLMM, including all 4 arousal types together, with a significant post hoc contrast when considering T–M+ and T+M– arousals versus early cortical Aβ burden (Table 2). Results were not driven by arousals occurring in non-REM (NREM) or REM sleep as statistical outputs were the same if we considered the 4 types of arousals in NREM/REM separately (M+ only in REM as arousal definition is REM requires change in muscle tone) (Table 2).

### Arousals linked with better Aβ status are associated with better cognitive performance.

**Figure 4:**
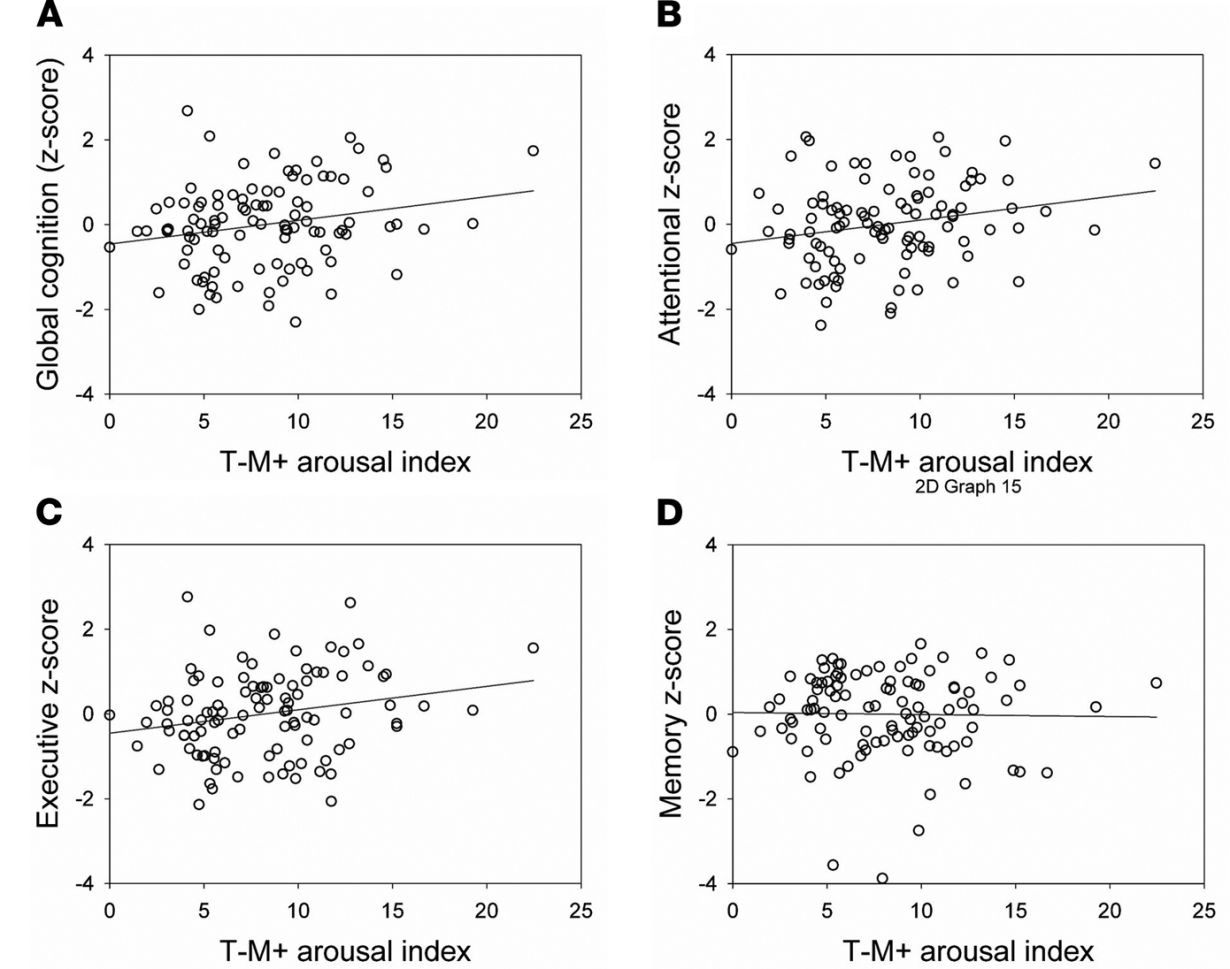
Association between T–M+ arousals prevalence and cognitive performance. (**A**) T–M+ arousals and global cognition (Spearman’s *r* = 0.21, *P* = 0.04); (**B**) T–M+ arousals and attention (Spearman’s *r* = 0.22, *P* = 0.03); (**C**) T–M+ arousals and executive functioning (Spearman’s *r* = 0.21, *P* = 0.03); (**D**) T–M+ arousals and memory (Spearman’s *r* = –0.03, *P* = 0.71). See Table 3 for full GLMM outputs. T, arousal associated (T+) or not (T–) with sleep stage transition; M, arousal associated (M+) or not (M–) with an increase in EMG tone. Indexes correspond to hourly prevalence.

We then tested whether cognition, as assessed in a global index through an extensive neuropsychological test battery, was differentially associated with the 2 arousal types showing opposite association with early cortical Aβ burden. In a GLMM, the association between global cognition and T–M+ arousal index was found to be significant (*P* = 0.048, *R*²β* = 0.04), on top of the education effect, but no relation with T+M– arousal index (*P* = 0.25), age, or sex (Table 3 and Figure 4) was found. Additional exploratory GLMMs with each specific cognitive domain, in turn, showed that this association was driven by the attentional domain (*P* = 0.032, *R*²β* = 0.047) and was not significant for the executive (*P* = 0.09) or memory domains (*P* = 0.91).

**Figure 5:**
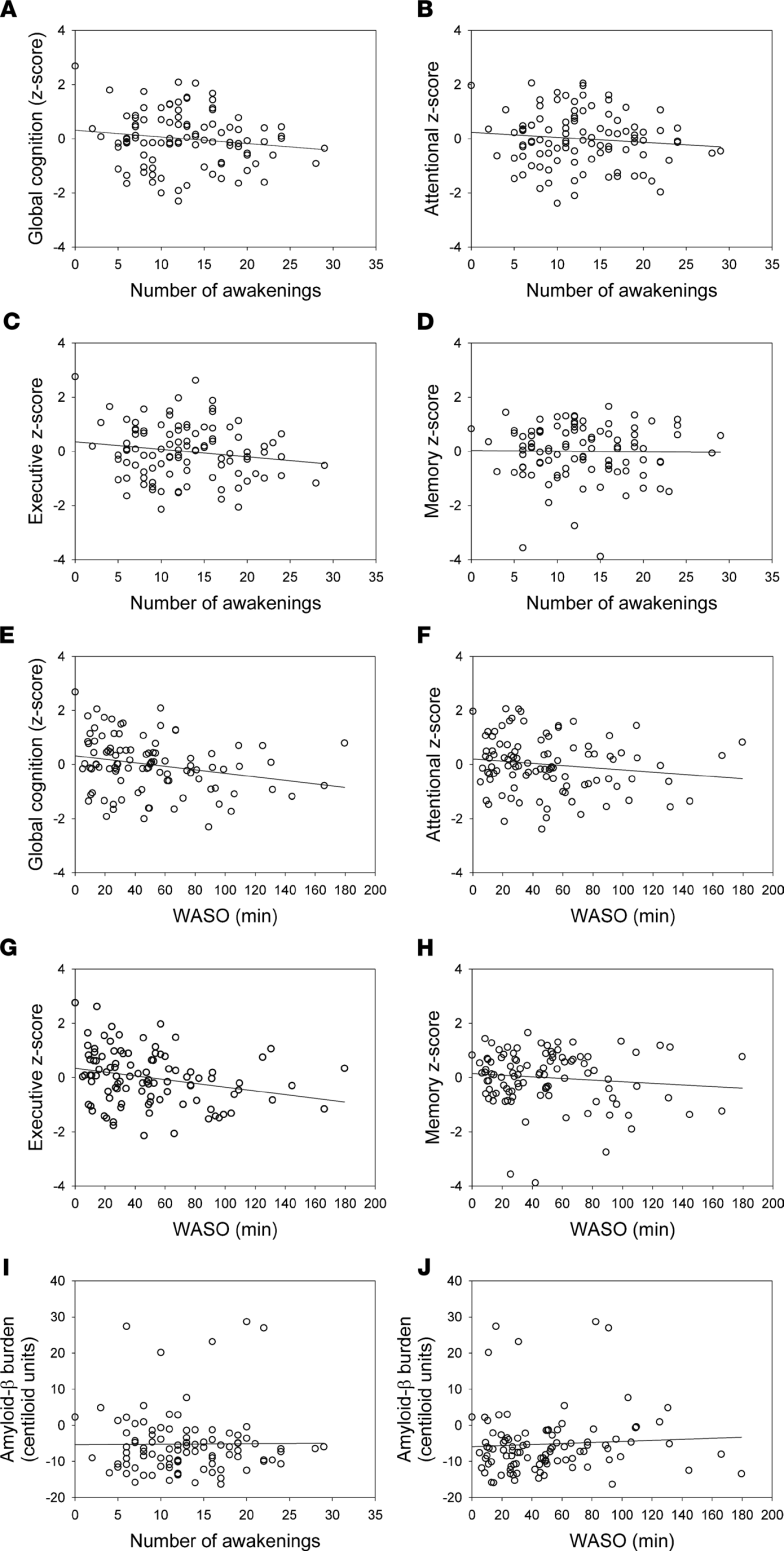
Association between number of awakenings and cognition. (**A**) Global cognitive performance (Spearman’s *r* = –0.10, *P* = 0.30; GLMM F_1,95_ = 2.21, *P* = 0.14); (**B**) attentional (Spearman’s *r* = –0.07, *P* = 0.48; GLMM F_1,95_ = 0.89, *P* = 0.35); (**C**) executive (Spearman’s *r* = –0.11, *P* = 0.25; GLMM F_1,95_ = 3.53, *P* = 0.06); (**D**) memory performances (Spearman’s *r* = –0.03, *P* = 0.80; GLMM F_1,95_ = 0.00, *P* = 0.98); (**E**) between WASO and cognition global cognitive performance (Spearman’s *r* = –0.24, *P* = 0.01; GLMM F_1,95_ = 4.66, *P* = 0.03); (**F**) attentional (Spearman’s *r* = –0.17, *P* = 0.11;GLMM: F_1,95_ = 0.56, *P* = 0.46); (**G**) executive (Spearman’s *r* = –0.28, *P* = 0.005;GLMM: F_1,95_ = 7.58, *P* = 0.007); (**H**) memory performances (Spearman’s *r* = –0.06, *P* = 0.59; GLMM: F_1,95_ = 1.11, *P* = 0.30); (**I**) between early cortical Aβ burden and number of awakenings (Spearman’s *r* = 0.01, *P* = 0.90; GLMM F_1,95_ = 0.22, *P* = 0.64); and (**J**) WASO (Spearman’s *r* = 0.11, *P* = 0.28; GLMM F_1,95_ = 0.05, *P* = 0.83).

We assessed the specificity of the findings for T–M+ arousals and considered the potential link between the number of full awakenings during sleep and wake after sleep onset (WASO) and the different cognitive measures in separate exploratory GLMMs. We found no link between cognition and the number of awakenings (Figure 5, A–D), while a significant negative association was detected between WASO and global cognition (F_1,95_ = 4.66, *P* = 0.03) which was driven by the executive domain (F_1,95_ = 7.58, *P* =.007) (Figure 5, E–H). Furthermore, neither WASO nor number of awakening was associated with early cortical Aβ burden (Figure 5, I and J).

## Discussion

Brain dynamics that buttress cerebral functions entail stationary and nonstationary interactions between neuronal populations (20). Sleep stages, which can be seen as enduring and widespread oscillatory modes sculpting brain activity, allow recurrent, brief, fast oscillatory activity, which sometimes leads to stage transitions (21, 22). Here, we focused on spontaneous arousals because their functional correlates remain undetermined. They are usually considered to induce sleep disruption and its detrimental functional consequences. However, spontaneous sleep arousals — i.e., not elicited by identifiable event — might also carry positive effects on brain functions. We quantified the prevalence of spontaneous arousals during undisturbed sleep in healthy individuals in late midlife and assessed whether it was associated with early cortical Aβ deposition and cognitive performance. Based on the theoretical concept that sleep arousals are diverse (11), we classified them according to their temporal association with a change in muscular tone and a sleep stage transition. These criteria were deemed clinically relevant, as arousals may or may not affect sleep macrostructure while muscular tone constitutes an arousal marker in REM. Based on this straightforward phenotyping in a large data sample, we provide the first empirical evidence that different types of sleep arousals have distinct correlates in terms of cognition and brain amyloid burden. Indeed, we found that arousals associated with sleep transitions (T+M–) are associated with higher cortical Aβ deposition in brain regions affected early on by AD neuropathology, suggesting their association with sleep fragmentation and worse brain status. By contrast, the more prevalent T–M+ arousals, which do not result in sleep transitions, are all the more frequent as Aβ deposition is low and cognitive performance superior, particularly in the attentional domain. This arousal type is therefore associated to a more favorable brain and cognitive status. Although sizes of the effects we detected remained modest, enduring small phenomenon can shape lifelong trajectories. The present findings may therefore be of particular importance since arousals have been reported to increase with age and since age represents the most important risk factor for cognitive decline and AD (2).

Our analyses show that the main characteristic differentiating the 2 types of arousals is whether or not they lead to a sleep stage transition. A second important criterion consisted of the concomitant increase in EMG tone. Aside from their different links with Aβ burden and cognition, T+M– and T–M+ arousals are not correlated with each other and differ in their spectral composition: T+M– bear a larger proportion of θ power, while T–M+ arousals are composed of a higher proportion of β power. The reason T–M– and T+M+ arousals are not significantly associated with Aβ and cognition is unclear and might reside in different prevalence or in diverging effects of sleep transitions and EMG bursts, which would hinder the relationship. Future studies are warranted to further investigate this issue.

Two hypotheses can be put forward to explain the heterogeneity in arousals. On the one hand, all arousals, triggered by a common set of brain areas, might be part of a continuum in which each arousal is characterized by the intensity in its driving neural activity, its spectral composition, its associated muscular tone, and its probability of sleep stage transition. Alternatively, the 2 arousal types are distinct physiological events prompted by different triggering brain structures and propagation cerebral networks. Oddly enough, the origin of spontaneous arousals remains elusive. Recent functional MRI (fMRI) data show that subcortical regions (including the thalamus, midbrain, basal ganglia, and cerebellum) were activated during non-REM arousals, while cortical regions were deactivated (23). A recent yet-to-be-reviewed study in rodents provides evidence that arousals leading to sleep state transition are at least partly driven by the locus coeruleus (LC), brainstem source of norepinephrine, which has a strong and ubiquitous influence on distant cortical brain regions, including during sleep (24). In addition, optogenetic stimulation of the LC causes immediate sleep-to-wake transitions, from both NREM and REM sleep, and results in high-frequency EEG activity (25). Therefore, subcortical activity — for instance, in the LC — could underlie transition arousals while no-transition arousals could also merely be the reflection of cortico-cortical or thalamo-cortical interplay (11). Identifying the brain sources of the 2 types of arousals would require invasive animal testing, coupling EEG to fMRI recordings in humans, or source reconstruction of high density EEG signals (22).

The cellular and molecular underpinnings of the distinct relationship between the 2 types of arousals, Aβ burden, and cognition are currently unknown. We can reasonably speculate that T+M– arousals have 2 potentially deleterious impacts. Firstly, they interrupt a sleep stage and consequently all its associated cellular phenomena, like plasticity (21). Secondly, it seems possible that they considerably increase cellular activity in diffused cerebral regions, a condition conducive to increase Aβ release. By contrast, one could tentatively speculate that T–M+ arousals promote Aβ clearance, hypothetically by increasing the pulsatility of cortical penetrating arteries (26). Additionally, T–M+ arousals might offer recurring opportunities to transiently synchronize distant brain areas, in frequency bands otherwise related to cognition during wakefulness (e.g., β oscillations; ref. 27) without enduringly disrupting the underlying brain oscillations (i.e., sleep state), similarly to what sleep spindles allow over σ band (12-16Hz) oscillations (28). In complex dynamics wordings, T–M+ arousals can be seen as distinct dynamics generated when the oscillatory trajectory is trapped in a local submanifold of an attractor (29), meaning the arousal would represent only a temporary breakout from the global oscillatory regime. These transient oscillations give rise to dynamic instability, despite the fact that the global manifold does not change. Dynamic instability is a form of complexity in neuronal systems that is critical for adaptive brain functions such as selection in self-organizing systems, learning, or memory (20). On the other hand, T+M– arousals would represent a distinct type of complexity, where the involvement of the brainstem would lead to a change in oscillatory regime through a change in the attractor manifold. Similar transient oscillations have been previously reported during wakefulness and have been reported to be related to cognition (20). Further studies are needed to unravel whether higher T+M–/lower T–M+ arousal indexes are facilitating Aβ aggregation or if, conversely, accumulating Aβ burden is disrupting sleep processes (2). Data in young individuals, in which current Aβ detection is typically negative (18), as well as longitudinal studies are needed to address this issue.

We emphasize that (a) our cohort only comprised healthy individuals, devoid of SDB, and (b) we focused on spontaneous arousals, which are not generated in response to detectable endogenous or exogenous perturbation (e.g., apnea or noise). Therefore, our findings probably do not apply to potentially more prevalent perturbation-induced arousals and their negative behavioral (15, 16) and neurodegenerative aftermaths (6). We also underline that we aimed to investigate links between sleep and Aβ burden early on in this neuropathological process; therefore, our volunteers did not show large Aβ deposition (only 5 could be considered as Aβ positive). Although Aβ is a hallmark of AD neuropathology, we do not know which volunteer will develop AD and, therefore, which one can be considered to undergo a true AD process — or an AD-like or AD-related process. As for any Aβ signal, its predictive value remains debated. It is tantalizing to suggest, and empirically testable, that arousals found in SDB mostly consist in transition arousals, which would contribute in part to the higher risk for AD reported in SDB (30). We further found no significant link between early Aβ burden and the number of full night-time awakenings during sleep or with WASO, 2 markers related to the fragmentation of sleep macrostructure defining in part sleep quality. The associations we find with Aβ burden in healthy late midlife appear, therefore, to be stronger with — if not specific to — sleep arousals, as compared with other indices of wakefulness during sleep or fragmentation of sleep. This contrast with a previous actigraphy study that reported correlations between WASO and Aβ burden in participants older than those included here (mean, 76.7 ± 3.5 years) (31). Our findings may therefore suggest that, at a younger age (~59 years), the detrimental association between sleep quality and AD neuropathology initially concerns transition arousals, leading to sleep macrostructure fragmentation, before being subsequently detected over other markers of sleep fragmentation.

Sleep arousals may connect the sleeper’s brain with the surrounding endogenous and exogenous relevant incoming information and contribute to elements of cortico-cortical information processing (11, 29), as done through sleep spindles, another fundamental feature of sleep microstructure (28). In other words, our findings suggest that sleep arousals, and their coalescence with other brain oscillations during sleep, may actively contribute to the beneficial functions of sleep. Arousals may interact with spindles and slow waves, however, so that we cannot rule out a contribution of these events to the effects we report. Future research should assess whether arousals are predictors of Aβ burden independent of other known neurophysiological elements/oscillations of sleep linked to cognition and brain health. Visual inspection of the data indicates that, despite occasional cooccurrence, slow wave arousals are not strongly nor systematically associated with any type of arousals. Our findings constitute the first empirical evidence of the conceptual existence of different arousal types differently associated to important parameters of cognitive and brain health (11). Sleep microfragmentation, as easily indexed by automatic detection of spontaneous arousals, could therefore potentially constitute a marker of favorable brain and cognitive trajectory in clinical practice, at least in late midlife adults and/or in individuals with still early AD-related neuropathology.

## Methods

### Study design and participants.

In order to target early Aβ brain deposit (18), we recruited healthy older individuals aged 50–70 years. In total, 208 volunteers were recruited, of which 101 participated in the actual study (Table 4). The rest were excluded due to one of the following exclusion criteria: clinical symptoms of cognitive impairment (dementia rating scale < 130; mini mental state examination < 27); BMI ≤ 18 and ≥ 29; recent psychiatric history or severe brain trauma; documented/diagnosed sleep pathologies such as insomnia and REM behavior disorder; medication affecting the CNS; smoking; excessive alcohol (>14 units/week) or caffeine (>5 cups/day) consumption; shift work in the past 6 months; or transmeridian travel in the last 2 months.

Participants were screened for sleep apnea/hypopnea syndrome during an in-lab night of sleep under polysomnography (PSG) preceding the one that was analyzed in the results section of this paper. This PSG included EEG (Fz, Cz, C3, PZ, Oz electrodes), 2 bipolar electrooculograms (EOGs), 2 bipolar submental EMG electrodes, 2 bipolar electrocardiograms (ECGs), 2 sets of bipolar leg electrodes, thorax and abdominal belts, an oximeter, a nasal canula, and a snoring sensor. As is typically done in similar sleep studies (7, 32), volunteers with an apnea/hypopnea index (AHI) ≥ 15/hour were excluded (79 subjects had an AHI ≥ 0 and < 5; 19 subjects had an AHI ≥ 5 and < 10; and 3 subjects had an AHI ≥ 10 and < 15). Given the low arousal index of our volunteers and the low rate of PLMS in our sample (9 subjects had a PLMS index ≥ 15), and given that controlling for those 2 covariates did not change the statistically significant associations we found in the reported models, we did not include them in the statistical analyses reported below. One volunteer was excluded from analyses that included Aβ data due to corrupted PET scan data caused by technical issues during acquisition. Demographic characteristics of the study sample can be found in Table 4.

### Sleep assessment.

Participants came to the lab for an adaptation night under PSG, after which those with sleep an AHI ≥ 15/hour were excluded from further participation. Volunteers were required to follow a regular sleep-wake schedule (±30 minutes) for 1 week based on their preferred bed and wake-up times before sleep EEG recording, in order to record their sleep in settings as close as possible to habitual conditions. Compliance was verified using sleep diaries and wrist actigraphy (Actiwatch, Cambridge Neurotechnology). Participants then joined the laboratory about 6.5 hours prior to habitual sleep time and were maintained in dim-light thereafter. Undisturbed habitual sleep was recorded with N7000 amplifiers (EMBLA, Natus) using 11 EEG derivations placed according to the 10–20 system (F3, Fz, F4; C3, Cz, C4; P3, Pz, P4; O1, O2 electrodes), 2 bipolar EOGs, and 2 bipolar submental EMG electrodes. Recordings were sampled at 200 Hz and rereferenced to the mean of the 2 mastoids.

### Arousal detection.

Sleep stage scoring and arousal detection were carried out in separate steps by 2 independent algorithms. Sleep stage scoring was performed in 30-second windows using a validated algorithm (ASEEGA, Physip) (33, 34). Automatic arousal detection was then computed as it is objective and reproducible, and because it saves time (35). We used an individually tailored validated algorithm based on the American Academy of Sleep Medicine (AASM) definition (12) of arousal but without using sleep stage information. Automatic scorings were visually inspected following computation.

In brief, arousal detection is performed over all electrodes on whole-night recordings split into 1-second epochs in 2 successive steps computed over the power in the broad-α (7–13 Hz), β (16–30 Hz), and lower-θ (3–7 Hz) frequency bands, excluding the σ band (11–16 Hz) — i.e., corresponding to frequency of sleep spindles — which cannot be considered as arousals. A fixed threshold is first applied to detect abnormal EEG activity relatively to the whole-night recording: any 1-second epoch with power in any of the 3 frequency bands higher than the whole-night median value in each frequency band is considered as a potential arousal. The second step adapts the threshold to account for the specific EEG background activity in a shorter time window. A specific threshold is computed for each 30-second window: all 1-second epochs without concomitant EMG tone increase are selected, as well as the first ten 1-second epochs without EMG increase before and after the 30-second window being evaluated; threshold of each frequency band consists in the median power over the selected 1-second epochs. Events composed of at least 3 consecutive 1-second epochs with changes in EEG frequencies higher than twice the local median and 1 median of the whole recording for that frequency band were considered as arousals. For detailed explanations on the method, see ref. 35.

In order to evaluate the potential heterogeneity of arousals, we split them according to 2 criteria, which we considered as relevant in research settings, as well as clinical practice. The first criterion addressed whether arousals did trigger a sleep stage transition (T+) (when they occurred within 15 seconds of a stage change — in the second half of an epoch preceding a stage change or in the first half of an epoch assigned a different stage than the previous epoch) or whether they did not (T–). The second criterion addressed their salience, reflected by the concomitant increase in EMG tone (M+) or its absence (M–).

Spectral analysis of arousals’ power was carried out through a time-frequency analysis on the first 3 seconds of arousals using Morlet’s wavelet transform in SPM12 (https://github.com/spm/spm12/, commit SPM12 r7219) on Fz electrode. Detrending was done over the 500 ms prior to the arousal event. Data were then averaged per arousal type prior to summing in the typical EEG bands that may compose an arousal (θ, 4.5–7.5 Hz; α, 8.5–11.5 Hz; and β, 16.5–29.5 Hz). Given the variety of factors that impact total power (e.g., conductivity of the involved tissues, such as scalp, skull, CSF) (36), and thus renders it complex to compare across subjects, relative power of each band was computed through a normalization relative to 0.5–30 Hz total power.

### MRI data.

MRI data were used in order to determine the region of interest used for extraction of Aβ burden value based on PET images. Quantitative multiparametric MRI acquisition was performed on a 3-Tesla MR scanner (Siemens MAGNETOM Prisma, Siemens Healthineers) to get a magnetization transfer–weighted (MT-weighted) contrast, based on multi-echo 3D fast low angle shot at 1 mm isotropic resolution (37) (with flip angle = 6° and application of additional off-resonance Gaussian-shaped RF pulse). MRI multiparameter maps were processed with the hMRI toolbox (38) (http://hmri.info) and SPM12 (Welcome Trust Centre for Neuroimaging, London, United Kingdom) to obtain a quantitative MT map and segmented images (gray matter, white matter, CSF), normalized to the standard MNI space using unified segmentation (39).

### PET scan.

Aβ PET imaging was performed using [^18^F]Flutemetamol, except for 3 volunteers for which [^18^F]Florbetapir was used. PET scans were performed on an ECAT EXACT+ HR scanner (Siemens). Participants received a single dose of the radioligand in the antecubital vein (target dose 185 MBq); images acquisition started 85 minutes after the injection and consisted of 4 frames of 5 minutes, followed by a 10-minute transmission scan using ^68^Ge line sources. Images were reconstructed using filtered back-projection algorithm including corrections for measured attenuation, dead time, random events, and scatter using standard software (Siemens ECAT, HR + V7.1, Siemens/CTI). Individual PET average images were produced using all frames and were then manually reoriented according to MT-weighted structural MRI volumes and coregistered to the individual space structural MT map. Flow-field deformation parameters obtained from DARTEL spatial normalization of the MT maps were applied to averaged coregistered PET images (40). We did not provide correction for partial volume effect, as this type of PET processing was not included in Centiloid scaling pipeline (41). Volumes of interest were determined using the automated anatomical labeling (AAL) atlas (42). Standardized uptake value ratio (SUVR) was computed using the whole cerebellum as a reference region (41). As images were acquired using 2 different radioligands, their SUVR values were converted into Centiloid units (41). Aβ burden was averaged over a composite mask covering the previously reported earliest aggregation sites for Aβ pathology (18) — frontal medial cortex and basal part of temporal lobe (fusiform and inferior temporal gyri).

### Cognitive assessment.

A cognitive battery of neuropsychological tasks was carried out in 2 sessions, while well rested. A first session of ~1 hour was performed in the afternoon prior to the sleep assessment, approximately 7.5 hours before habitual bedtime, and a second session of ~1.5 hours was performed on another day (between 12 and 6 hours prior to habitual bedtime). From those 2 sessions, 3 domain-specific composites scores were computed for the memory, executive function, and attentional domains, and they consisted of the standardized sum of the standardized domain-specific scores, where higher values indicate better performance. A fourth global cognitive score consisted of the standardized sum of the domain-specific composite scores.

The first session comprised (a) mnemonic similarity task (MST) (43); (b) category verbal fluency (letter and animals) (44); (c) digit symbol substitution task (DSST) (45); (d) visual N-back task (1-, 2-, and 3-back variants) (46); and (e) choice reaction time (CRT) (47). The second session of ~1.5 hours was performed on another day (between 12 and 6 hours prior to habitual bedtime) and comprised (a) direct and inverse digit span task (45); (b) free and cued selective reminding test (FCSRT) (48); (c) a computerized version of the Stroop test (49); (d) trail making test (TMT) (50), and (e) D2 attention test (51).The memory score consisted of the FCSRT (sum of all 4 free recalls) and the recognition memory score from the MST. The executive function score included verbal fluency tests (letter and animals score for 2 minutes), inverse order digit span, TMT (part B), N-back (3-back variant), and Stroop test (interfering item errors). The attentional score comprised the DSST, TMT (part A), N-back (1-back variant), D2 (Gz-F), and CRT (reaction time to dissimilar items).

### Statistics.

Statistical analyses were performed in SAS 9.4 (SAS Institute) using GLMMs. The distribution of dependent variables was determined by fitting all parametric probability distributions to data, using the “allfitdist” function in Matlab 2015 (http://amir.eng.uci.edu/MvCAT.php), and GLMMs were adapted accordingly as preconized by SAS statisticians. A subject was treated as a random factor (intercept); each model included sex and age as covariates, as well as education for models with cognitive score as dependent variables. Statistical significance threshold was set at *P* < 0.05 as no correction for multiple comparisons were required. The association between arousals and Aβ was tested in a single model, including arousal density as dependent variable together with transition (T+, T–) and EMG (M+, M–) arousal statuses and early Aβ burden as regressors (as well as sex and age). The association between arousal and cognition was tested in a single model including global cognition score as a dependent variable together with T+M– and T–M+ arousal density as regressors (as well as sex and age). Kenward-Roger correction was used to determine degrees of freedom. *R*²β* values were computed to estimate the effect sizes of significant fixed effects and statistical trends in all GLMMs (52). *P* values in post hoc contrasts (difference of least square means) were adjusted for multiple testing using Tukey’s procedure. Cook’s distance was used to assess the potential presence of outliers driving the associations, and as values ranged below 0.4, no data point was excluded from the analyses (a Cook’s distant > 1 is typically considered to reflect outlier value).

### Study approval.

The study was registered with EudraCT 2016-001436-35. All procedures were approved by the Hospital-Faculty Ethic Committee of ULiège. All participants signed an informed consent prior to participating in the study.

## Author contributions

Study concept and design were contributed by ES, PM, CP, C Bastin, FC, and GV. Data acquisition, analysis, and interpretation were contributed by DOC, MVE, JN, MG, EK, C. Berthomier, PB, MB, ES, MAB, C Bastin, FC, CP, PM, VM, and GV. DOC and GV drafted the first version of the manuscript. All authors revised the manuscript and had final responsibility for the decision to submit for publication. While all co–first authors tightly collaborated to acquire and analyze the data for an equivalent time and workload, each of the co–first authors had their own aspect of the data to deal with in priority. The order reflects these priorities.

## Supplementary Material

ICMJE disclosure forms

## Figures and Tables

**Table 4:**
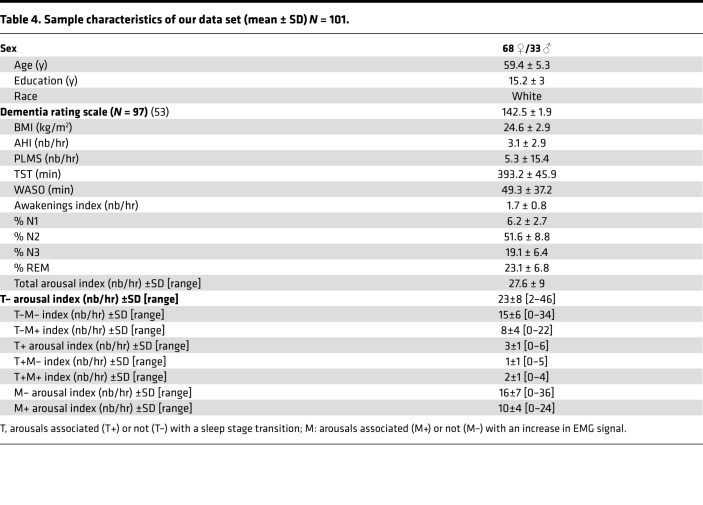
Sample characteristics of our data set (mean ± SD) *N* = 101.

**Table 3:**
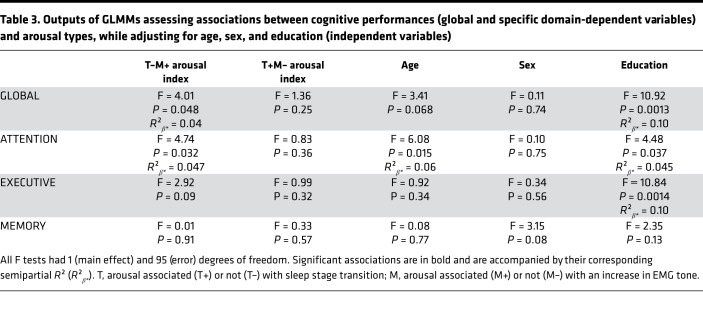
Outputs of GLMMs assessing associations between cognitive performances (global and specific domain-dependent variables) and arousal types, while adjusting for age, sex, and education (independent variables)

**Table 2:**
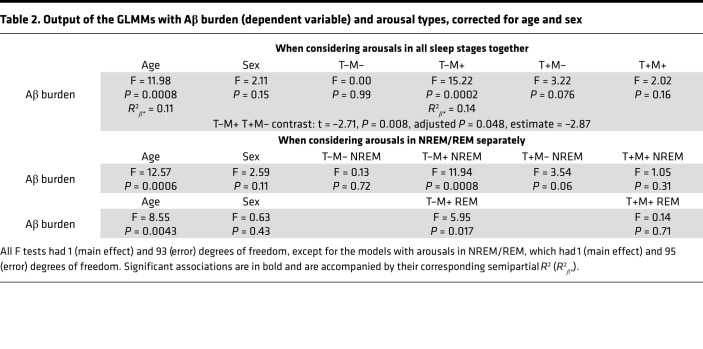
Output of the GLMMs with Aβ burden (dependent variable) and arousal types, corrected for age and sex

**Table 1:**
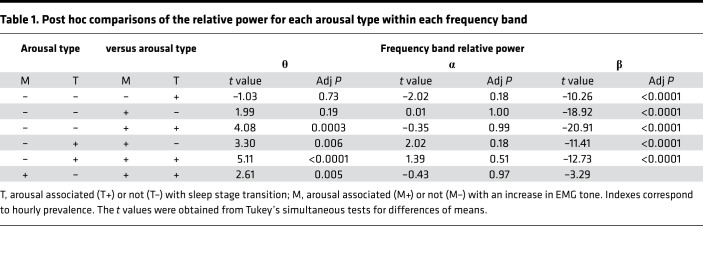
Post hoc comparisons of the relative power for each arousal type within each frequency band

## Version 1 11/16/2021

In-Press Preview

## Version 2 12/22/2021

Electronic publication
